# Wide Two-Degree-of-Freedom Static Laser Scanner with Miniaturized Transmission Mechanism and Piezoelectric Actuation

**DOI:** 10.3390/s21186077

**Published:** 2021-09-10

**Authors:** Takashi Ozaki, Norikazu Ohta, Motohiro Fujiyoshi

**Affiliations:** Toyota Central R&D Labs. Inc., Nagakute 480-1192, Japan; ohtan@mosk.tytlabs.co.jp (N.O.); fujiyoshi@mosk.tytlabs.co.jp (M.F.)

**Keywords:** MEMS, optical actuator, piezoelectric actuator, scanning mirror

## Abstract

In recent years, laser scanners have attracted significant attention for applications such as laser radars. However, the establishment of a two-degree-of-freedom scanner that can quasi-statically drive a large mirror with a large deflection angle has proven to be challenging. In this paper, we propose a laser scanner design and fabrication method by combining two unimorph piezoelectric actuators composed of piezoelectric single-crystal Pb(In_1/2_Nb_1/2_)O_3_-Pb(Mg_1/3_Nb_2/3_)O_3_-PbTiO_3_ and a miniature translation-rotation conversion mechanism with flexible polyimide hinges. The size of the entire scanner was 32 mm × 12 mm × 10 mm. We successfully demonstrated that the scanner could achieve a large quasi-static mechanical deflection angle amplitude of 20.5° in two axes with a 6-mm-square mirror.

## 1. Introduction

Laser scanners, which are realized by maneuvering a movable mirror using an actuator, are expected to have a wide range of applications, including displays [1,2,3,4], laser radars [5,6,7], optical communications [8,9], and laser processing [10,11]. A wide scanning range is desirable in all these applications. To achieve this, increasing the scan amplitude through resonance is a simple and effective method; so resonant-driven scanners have been described in many papers. However, resonance driving is not suitable for all applications. Consequently, there is a great demand for quasi-static vector scan driving in applications such as measurements based on laser radars, free-space optic communications, and laser processing.

The key parameters of laser scanners are mirror size, scan angle, and scan speed. As such, we focus on a time-of-flight (ToF) laser radar system. Compared to the ToF 3D camera based on a focal plane array [12], laser scanners have an advantage in terms of their signal-to-noise ratio owing to their ability to focus the laser beam on a single measurement point. Because the ToF measurement accuracy is determined by the amount of detected light, a larger mirror is desirable to collect smaller quantities of reflected light. The scan speed is also important because it determines the frame rate. Conventionally, laser scanning is performed using large, heavy, and expensive rotating mirrors powered by electromagnetic motors [13]. There is a great demand to replace them with smaller and less expensive actuators. Many small scanners based on micro-electro-mechanical systems (MEMS) technology have been studied, but most of them are small mirrors with diameters of less than 1 mm [14]. This is because the cost of fabrication using a semiconductor process increases dramatically when the chip size becomes larger. In addition, the dynamic deformation increases for thin-film mirrors. In other words, developing a scanner that can satisfy the criteria of large size, large displacement, and high speed in a miniaturized form remains a challenging.

Electrostatic [15,16,17,18], electrothermal [19,20,21,22], electromagnetic [23,24,25,26,27,28], and piezoelectric [29,30,31] forces have been reported as actuation alternatives for miniaturized laser scanners. Among these, electrostatic, electromagnetic, and piezoelectric are suitable for driving large mirrors rapidly owing to their high response speeds. Mirrorcle Inc. developed electrostatic scanners with large mirrors and wide deflection angles—that is, a 5-mm-circle mirror with a mechanical deflection angle of 10.4° [18]. A research group at Sogang University reported an electromagnetic scanner with an 8-mm-square mirror and a deflection angle of 8.0° [27].

However, piezoelectric scanners with both larger mirrors (≥5 mm) and wider deflection angles (≥10°) are yet to be demonstrated. Piezoelectric scanners can be classified into two types: small scanners (less than 1 mm) made using MEMS technology, and large mirrors driven by stacked piezoelectric actuators. Scanners with amplification mechanisms tend to have been studied [11,32] because the displacement of stacked actuators is small. However, only a small displacement angle—that is, less than a few degrees—has been achieved thus far. Moreover, stacked actuators have the disadvantage of high power consumption owing to their large capacitance.

Consequently, we propose a miniature two-degree-of-freedom (2-DoF) piezoelectric laser scanner that satisfies both the large mirror and wide scanning angle requirements—that is, a mechanical scanning angle of more than 20° using a 6 mm square mirror. This performance is significantly better than that of previous piezoelectric scanners, which. suggests the possibility of expanding the applicability of piezoelectric scanners.

## 2. Methods: Design

### 2.1. Structure of Developed Laser Scanner

**Figure 1:**
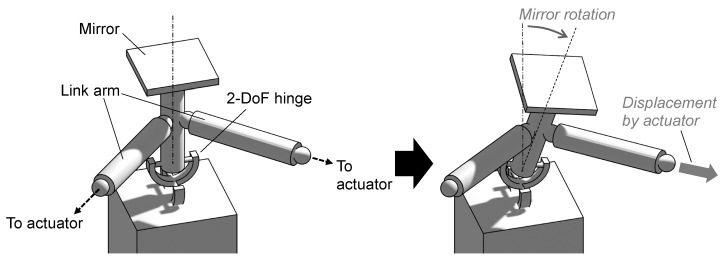
Concept of 2-DoF translation-rotation conversion mechanism.

Our scanner is equipped with two piezoelectric actuators. Each actuator tilts the mirror in a perpendicular direction to achieve a 2-DoF scan. Because the output displacement of the actuators is almost translational, a transmission mechanism exists between the actuators and the mirror to convert it into rotation. First, we describe the 2-DoF translation-rotation conversion mechanism. Figure 1 shows a simplified concept of it. There is a 2-DoF hinge at the bottom, and a mirror attached above it. There are two link arms extending between the hinge and the mirror in two directions. Each end of the link arm is connected to an actuator. When the link arm is pulled by the actuator, the mirror tilts in its direction (Figure 1, right).

**Figure 2:**
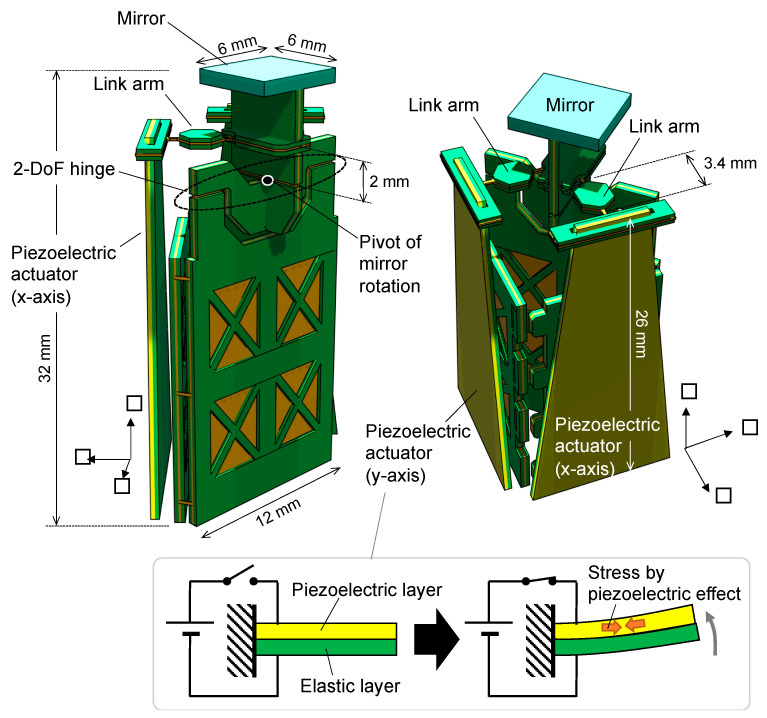
Structure of fabricated scanner.

Figure 2 shows the specific structure of the scanner. Its overall length is 32 mm along the longest direction. The conversion mechanism shown in Figure 1 corresponds to the upper half of the structure. We employed a bending unimorph configuration as the actuator, composed of a lamination of a piezoelectric and elastic layer. The stresses caused by the piezoelectric effect bends the actuator, as shown in the lower illustration of Figure 2. For the piezoelectric material, we used single-crystalline Pb(In_1/2_Nb_1/2_)O_3_-Pb(Mg_1/3_Nb_2/3_)O_3_-PbTiO_3_ (PIN-PMN-PT), which has a large piezoelectric constant (d_32_ = −1156 pm/V [33]) approximately five times larger than that of ordinary Pb(Zr,Ti)O_3_ (PZT). This unimorph configuration and the use of piezoelectric single crystals are one of the technical keys to our scanner. This supports a piezoelectric actuator with a large displacement output in the order of millimeters, which facilitates the realization of a wide scanning angle. The distance from the 2-DoF hinge (i.e., the pivot of mirror rotation) to the link arms was designed to be 2 mm. The length of the link arm is 3.4 mm. The piezoelectric actuators have a trapezoidal shape with a length of 26 mm. Two actuators are attached to drive the *x*- and *y*-axis directions, as described above. The mirror was made from single-crystalline silicon with a thickness of 0.5 mm.

**Figure 3:**
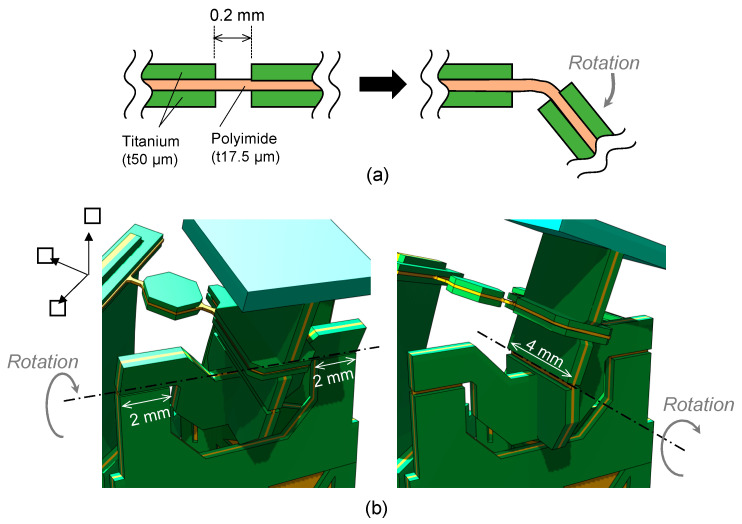
2-DoF hinge structure. (**a**) Cross-sectional image of hinge, and (**b**) 2-DoF hinge design and its motion.

**Figure 4:**
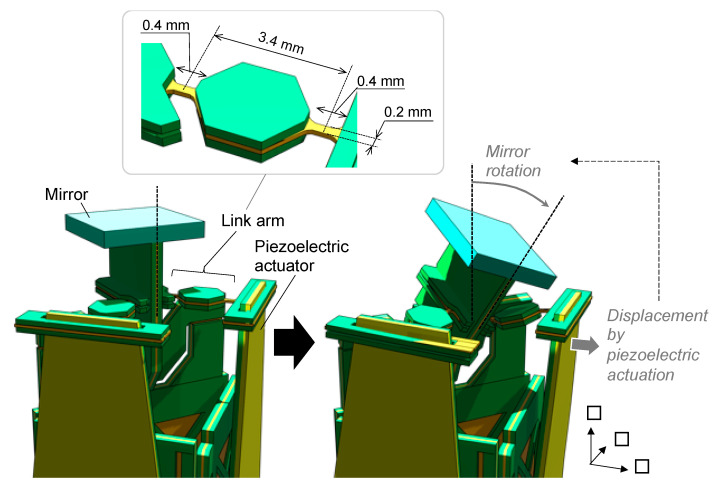
Actuation behavior and structure of link arm.

The structure of the 2-DoF hinge is shown in detail in Figure 3. Our mechanism consists of a thin polyimide (PI) sheet sandwiched between two titanium layers. As shown in Figure 3a, the hinge is realized by removing the titanium layers and leaving only the PI layer. The thicknesses of the titanium and PI are 50 μm and 17.5 μm, respectively, and their Young’s moduli of titanium and PI are approximately an order of magnitude different (110 GPa and 9 GPa, respectively). The thickness of the three-layered section is 6.7 times that of the single-layered PI section. Consequently, the bending stiffness is $10\times 6.7^{3}≃3000$ times greater in the triple-layered section than in the single-layered PI section. Thus, the hinge is close to an ideal joint with a very low stiffness compared to the other parts. The 2-DoF hinge was realized using a combination of two hinges along different axes, as shown in Figure 3b. Figure 4 shows the structure of the link arm and its behavior during actuation. Both ends of the link arm are equipped with a PI hinge (Figure 4, top). The thickness of the PI was designed to be 50 μm, to increase the rigidity of the hinge so that the link arm does not buckle when a compressive force is applied to it. When the piezoelectric actuator pulls the link arm, the mirror rotates, as shown in the lower portion of Figure 4.

### 2.2. Performance Analysis

**Figure 5:**
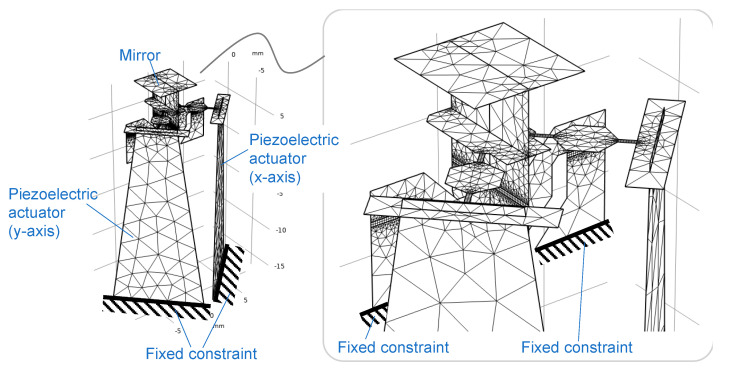
FEA model for resonant mode and static actuation angle.

In this section, we present the performance of the developed scanner based on finite element analysis (FEA) and a simplified theoretical model. Figure 5 shows an overview of the FEA model used in this study. Because our scanner was composed of thin plate structures, we constructed the model entirely using shell elements. The piezoelectric driving force was simulated by applying a bending moment corresponding to the piezoelectric stress to the elements of the actuator. COMSOL Multiphysics (COMSOL, Inc., Burlington, MA, USA) was used for our analyses. The material properties and calculation method of the values of the surface density, bending stiffness, and bending moment of the piezoelectric actuation adopted in this study are shown in Appendix A. We analyzed the resonant frequencies and static actuation characteristics (the relationship between the applied voltage and static deflection angle of the mirror) using FEA.

**Figure 6:**
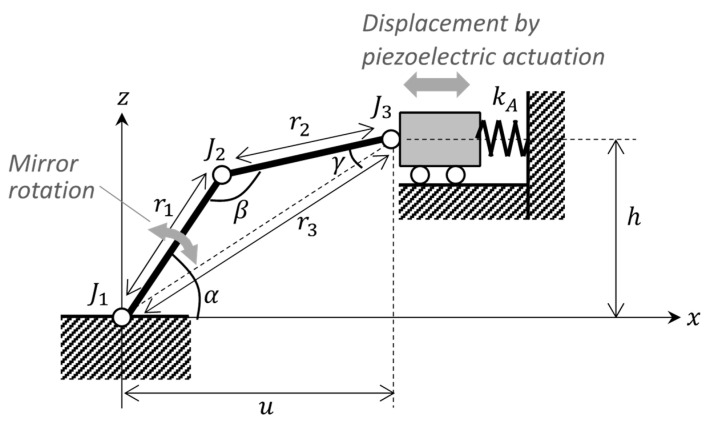
Simplified theoretical model to estimate static actuation angle.

We also derived the static actuation characteristics based on a simplified theoretical link-joint model, as shown in Figure 6. Here, only the actuation direction was considered, and the model was two-dimensional. The 2-DoF hinge corresponds to *J*_1_, and the link arm is the segment between joints *J*_2_ and *J*_3_. The deflection angle of the mirror is represented by a change in α. The piezoelectric actuator was modeled as a translational spring with spring constant $k_{A}$.

From the geometrical relationship, the angles of joints α, β, and γ are expressed as:

(1) $$\begin{array}{c} \alpha =\arcsin\frac{h}{r_{3}}+\arccos\left(\frac{r_{1}^{2}+r_{3}^{2}-r_{2}^{2}}{2r_{1}r_{3}}\right)\\ \beta =\arccos\left(\frac{r_{1}^{2}+r_{2}^{2}-r_{3}^{2}}{2r_{1}r_{2}}\right)\\ \gamma =\arccos\left(\frac{r_{2}^{2}+r_{3}^{2}-r_{1}^{2}}{2r_{2}r_{3}}\right) \end{array}\},$$

where $r_{1},\;\;r_{2}$ and $r_{3}$ are the distances between $J_{1}–J_{2}$, $J_{2}–J_{3}$ and $J_{3}–J_{1}$, respectively. $r_{3}$ can be rewritten using the height of $J_{3}$, h, and the position of the actuator from $J_{1}$, u, as $r_{3}=\sqrt{u^{2}+h^{2}}$. The joints can be approximated as linear springs. Thus, the potential energy stored in the joints and actuator U is given by:

(2) $$U=\frac{1}{2}k_{1}{\left(\alpha -\alpha_{0}\right)}^{2}+\frac{1}{2}k_{2}{\left(\beta -\beta_{0}\right)}^{2}+\frac{1}{2}k_{2}{\left(\gamma -\gamma_{0}\right)}^{2}+\frac{1}{2}k_{A}{\left(u-u_{0}-\delta_{A}\left(V\right)\right)}^{2},$$

where $k_{1},\;\;k_{2}$ and $k_{3}$ are the spring constants of joints $J_{1–3}$, respectively. $\alpha_{0},\;\;\beta_{0}$ and $\gamma_{0}$ are the initial angles. $\delta_{A}\left(V\right)$ is the no-load deflection of the actuator at an applied voltage V. We assume that the no-load actuation displacement is proportional to V: $\delta_{A}\left(V\right)=GV$, where G is a constant parameter. The static equilibrium state can then be obtained by solving:

(3) $$\frac{\partial U}{\partial u}=0.$$

Numerically solving Equation (3) leads to parameters for our scanner design, as summarized in Table 1. The derivations are described in Appendix B.

**Figure 7:**
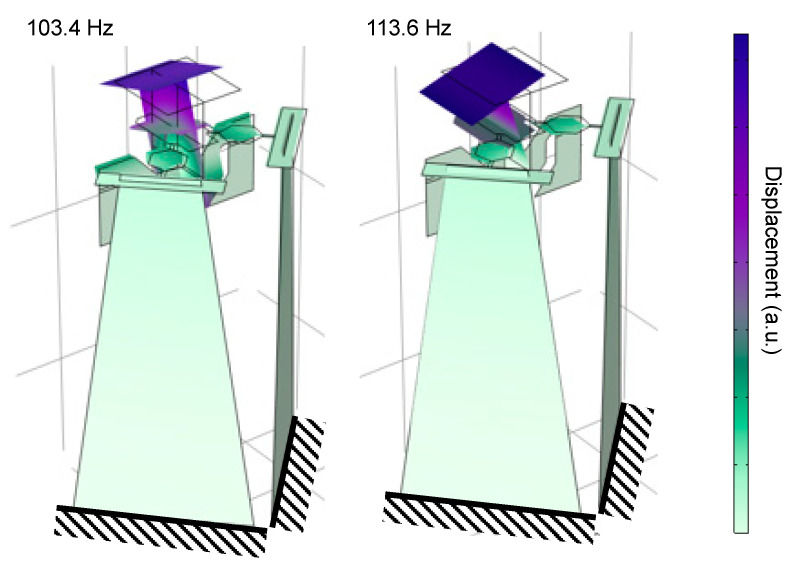
Resonant modes of proposed scanner.

**Figure 8:**
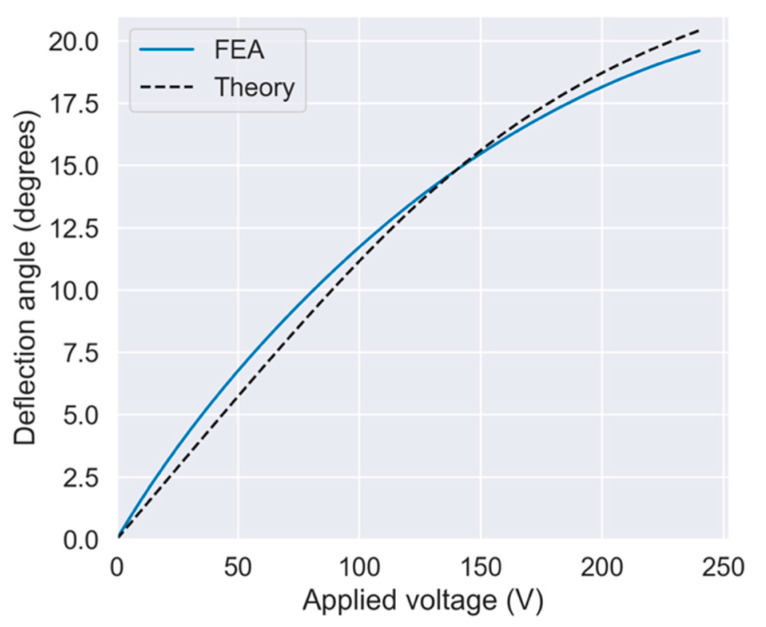
Relationship between applied voltage and deflection angle.

Next, we present the analytical results obtained using the models described above. Figure 7 shows the resonant modes corresponding to the scanning motions calculated by FEA; the resonant frequencies in the two axis directions were 103.4 and 113.6 Hz, respectively. Figure 8 shows the analytical results for the quasi-static actuation characteristics. In the graph, the solid blue and dashed black lines show the results of the FEA and simplified theoretical calculations, respectively. Because the FEA and theory were close to each other, it was suggested that our design was close to an ideal link-joint mechanism. Moreover, from this analysis a large deflection angle of approximately 20° was expected with an applied voltage of 240 V. The output deflection angle exhibited a large nonlinearity with respect to voltage—that is, as the voltage increased the output deflection angle increased more gradually. This was caused by geometrical nonlinearity which can be qualitatively interpreted as follows: As the displacement of the actuator increases, joints $J_{1-3}$ become closer to being in a straight line. In such a situation, the reaction force due to the spring stiffness of the joints increases for the actuator, making it more difficult for the actuator to displace the link arm.

### 2.3. Materials and Fabrication

In this section, we describe the fabrication method of our proposed scanner. The titanium layer was processed by wet etching, and the PI sheet was patterned by laser processing. We employed a PI sheet product with a thermally bonded layer (UPILEX-VT; Ube Industries, Ltd., Tokyo, Japan). Consequently, no additional adhesive was used, and the three layers were bonded by hot lamination. Wet etching of the titanium layer was performed by Hirai Seimitsu Kogyo Co. Ltd. (Osaka, Japan), and laser processing of the PI sheet was performed by Nano-Process Co. Ltd. (Hamamatsu, Japan). The detailed conditions of the hot lamination process are described in a previous report [34].

**Figure 9:**
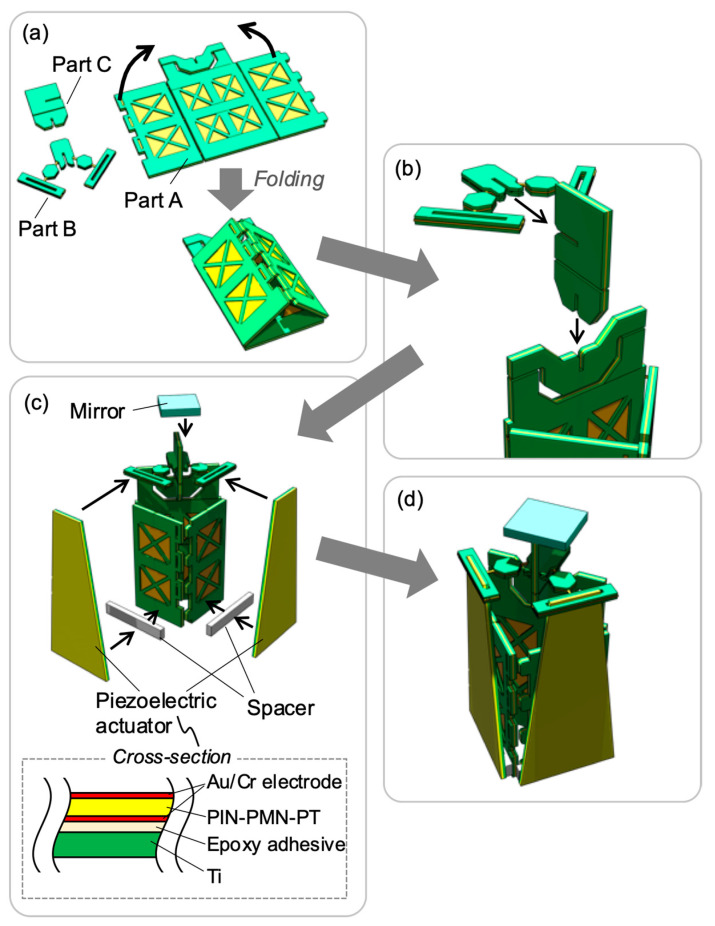
Assembly procedure of 2-DoF actuation mechanism: (**a**) origami assembly of Part A; (**b**) 2-DoF hinge assembly; (**c**) attachment of mirror and piezoelectric actuators; (**d**) completed device.

Figure 9 illustrates the assembly procedure. The transmission mechanism is divided into three parts. One of the parts includes half of the 2-DoF hinge and the base of the scanner (referred to as Part A), another includes the link arms (referred to as Part B), and the last part includes the remaining half of the 2-DoF hinge and the mirror fixing part (referred to as Part C), as shown in Figure 9a. Part A is folded like an origami and assembled into a triangular prism. The base rigidity is increased by this folding process. Next, the actuator is fixed to two orthogonal sides of the triangular prism; the displacement direction of the actuator being defined by the prism shape. Figure 9b shows the assembly of the 2-DoF hinge and the link arm. These parts are assembled by insertion into the slits on each side. A UV-curable adhesive (Loctite 4305; Henkel AG & Company, Düsseldorf, Germany) was used for fixation. The 2-DoF hinge is formed by inserting Part B into the top of Part A. The link arm is then attached by inserting Part C into Part B. Next, the mirror and actuators are attached, as shown in Figure 9c. The mirror is attached to the top of part C using a UV-curable adhesive. The actuators are fixed to the bottom of Part A with 2-mm-thick acrylic spacer blocks between them. All assembly processes were performed manually (by hand). The piezoelectric material PNI-PMN-PT was fabricated by cutting a bulk single crystal wafer material, manufactured by TRS Technologies, Inc. (State College, PA, USA). Au/Cr electrode was formed by sputtering on both sides of the PIN-PMN-PT plate. We employed titanium as the elastic layer of the unimorph actuator. The piezoelectric and elastic layers were bonded by epoxy adhesive (Quick 5; Konishi Co., Ltd., Osaka, Japan) whose thicknesses were 0.10 and 0.13 mm, respectively. 

## 3. Results and Discussion

**Figure 10:**
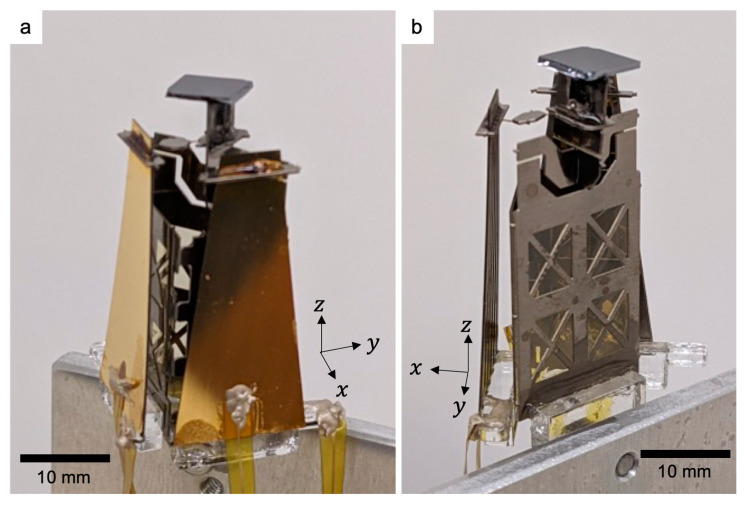
Photograph of fabricated scanner: (**a**) and (**b**) show the identical device viewed from different angles.

**Figure 11:**
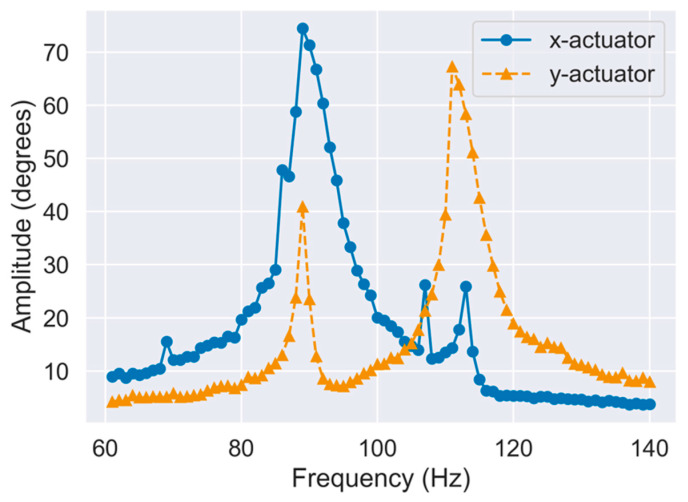
Frequency response of fabricated scanner.

A sinusoidal voltage with an amplitude of 3 V was applied between 60 and 140 Hz, and the deflection angle amplitude was measured. Photographs of the fabricated scanner are shown in Figure 10. The evaluation setup is presented in Appendix C. The resonance frequency was determined from the peak of the frequency–amplitude curve. The results are shown in Figure 11, where the amplitude peaks of 89 and 111 Hz were obtained for the *x*-axis and *y*-axis actuators, respectively. These results were in good agreement with FEA results described above, suggesting that a geometry close to the design was successfully fabricated. 

**Figure 12:**
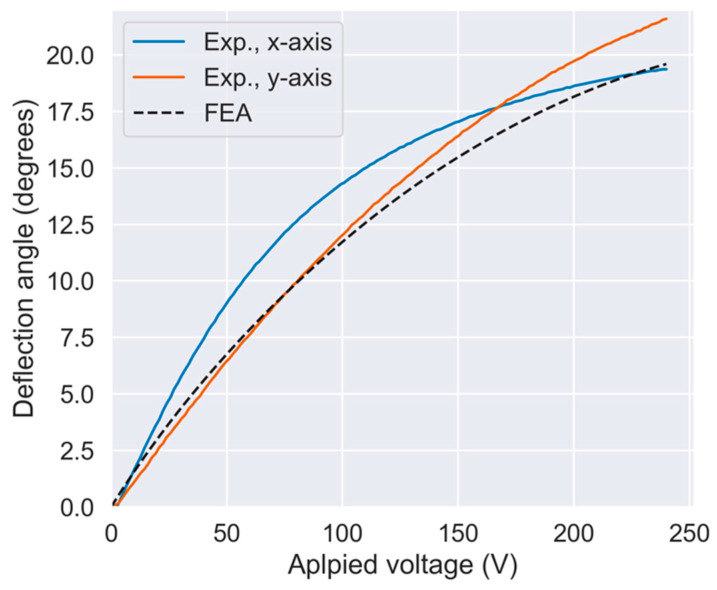
Measured quasi-static actuation characteristics.

Figure 12 shows the measurement results of the quasi-static actuation characteristics. Figure 12a,b show the results of the *x*-axis and *y*-axis actuation, respectively. In this graph, the solid lines show the measurement results, and the dashed lines show the FEA results. The solid blue and red lines represent the deflection angles of the *x*-axis and *y*-axis actuators, respectively. The applied voltage was set to a maximum of 240 V in this experiment. The average mechanical deflection angle at the maximum voltage was 20.5° (21.6° and 19.4° in the x- and y-directions, respectively), the measured characteristics agreeing well with the FEA results. 

The characteristics of the scanner were different in the x- and y-directions. We believe that this difference was due to manufacturing variations. Because the assembly was performed manually, the orthogonality of the link arms and the position of the actuator tips are likely to have exhibited large variations. The obvious difference in characteristics between simulated and real devices signifies a large manufacturing variability in production. Thus, improving the design to reduce the assembly error will be necessary. To improve this, it is important to reduce the number of parts and also incorporate an auxiliary structure for accurate positioning the assembly (which can be detached and discarded after assembly). It is also promising to incorporate a design such as the pop-up book MEMS proposed by Whitney et.al [17].

**Figure 13:**
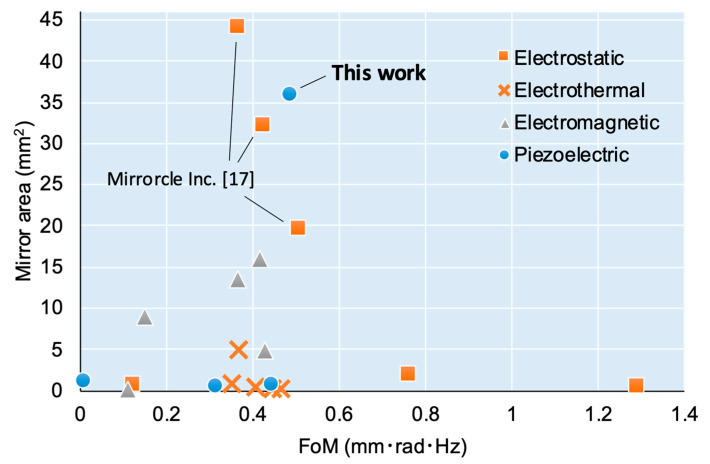
Comparison of 2-DoF quasi-static scanners.

To objectively compare our results, the performance of previous 2-DoF quasi-static scanners is summarized in Table 2 and Figure 13 and compared with our scanner. Here, we adopt the figure-of-merit (FoM) proposed in a review report [14] to be the scanner performance. The FoM is calculated as $\theta \sqrt{4A/\pi }f$, where θ, A, and f are the optical scanning angle amplitude, area of the mirror plate, and resonant frequency, respectively. Figure 13 shows a graph of the FoM on the horizontal axis and the mirror area on the vertical axis. The type of marker indicates the difference in actuation principle. There are only examples with small mirrors (less than 2 mm^2^) in the region of FoM≥0.5. The only scanners with a large area mirror and high FoM are the electrostatic products manufactured by Mirrorcle Inc. [18]. Our scanner exhibited better characteristics than the others, achieving noteworthy performance compared to previous cases. 

**Figure 14:**
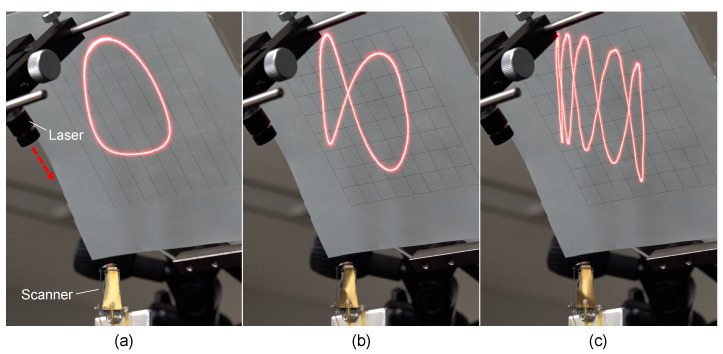
2-DoF scan demonstrations. (**a**) Circle, and Lissajous curves with the frequency ratio of (**b**) 2 and (**c**) 5.

Figure 14 shows the results of two-dimensional scans. Figure 14a–c show the results of using sinusoidal voltages with frequency ratios of 1, 2, and 5, respectively, to the piezoelectric actuators. The trajectories of the laser were obtained by superimposing multiple captured images. The Lissajous figures were successfully drawn, although they were distorted owing to a large nonlinearity in the relationship between the voltage and displacement angle, as shown in Figure 12.

## 4. Conclusions

We proposed a new laser scanner based on a combination of piezoelectric single-crystalline PIN-PMN-PT unimorph actuators and a 2-DoF transmission mechanism with a PI hinge. Experimentally, we succeeded in realizing a scanner that could scan a 6 mm square mirror with a quasi-static mechanical angle of more than 20° at an average resonant frequency of 100 Hz. 

However, three main limitations to the current design exist. The first is the high assembly cost and variations, as stated in Section 3. The experimental results showed a large variation in the actuation characteristics in the x- and y-directions. Therefore, a uniform and reproducible assembly structure and fabrication process will be considered in future work. The second is the high material cost of PNI-PMN-PT. Currently, this single crystal material is much more expensive than common PZT ceramics. Although future cost reduction can be expected, a design that can achieve the same performance as that of PZT ceramics is desirable. The third is the durability of the PI hinge. We did not observe any failures at least in this experiment. PI is the most suitable choice for polymer hinges due to its excellent durability among polymers; according to the specification provided by the manufacturer, the UPILEX used in this project can withstand more than 100,000 cycles of continuous bending. However, this is still a small number for laser scanner, and the reliability is uncertain compared to those of ordinary MEMS materials such as single crystal Si. The reliability should be verified in future work. 

Further improvements include dealing with nonlinearities and the resonant frequency. The closer its performance is to being linear, the easier it would be to control the deflection angle. A resonant frequency of about 100 Hz is too low for applications such as Lidar, industrial lasers, and displays. It can be promising for applications such as optical communications that do not require frequent switching and medical laser processing machines where miniaturization is more important than speed; however, these applications are limited. High speed (high frequency) is important to improve the versatility. Consequently, it would be desirable to design a higher resonance frequency without diminishing the performance of other parameters. In addition, many applications require laser pointing in precise directions. This requires the integration of a tilt angle sensing function. This can be achieved by patterning sensing electrodes in the piezoelectric unimorph and estimating the unimorph displacement from the output, as reported by Kobayashi et al. [35] and Zhang et al. [36]. Alternatively, strain gauges could be attached to the piezoelectric unimorph [37].

## Figures and Tables

**Table 1:** Parameters for simplified theoretical model.

Parameter	Value	Unit
$r_{1},\;r_{2},\;r_{3}$	2.72, 3.40, 5.62	mm
$k_{1},\;k_{2},\;k_{3}$	8.04, 4.69, 4.69	$10^{-5}$ Nm/rad
$k_{A}$	167	N/m
G	$5.27\times 10^{-3}$	mm/V

**Table 2:** Characteristics of 2-DoF quasi-static scanners.

Ref.	Actuation Principle	Mirror Area (mm^2^)	Deflection Angles (degrees)	Resonant Frequencies (Hz)	FoM (mm·rad·kHz)
[15]	Electrostatic	0.50	12.0	3800, 3900	1.29
[15]		2.0	12.0	670, 1600	0.76
[16]		0.79	12.4, 8.2	350, 320	0.12
[18]		19.6	10.4	278	0.50
[18]		32.2	5.56, 5.64	339, 337	0.42
[18]		44.2	2.49, 2.49	559, 557	0.37
[19]	Thermal	0.52	20.0	690, 740	0.41
[20]		0.20	2.0	12800	0.45
[21]		0.81	18.0	550	0.35
[22]		5.0	7.5, 6.0	615	0.37
[19]		0.25	51.0, 39.5	170, 870	0.46
[23]	Electromagnetic	4.9	30.0, 23.0	160, 210	0.43
[24]		9.0	10.0	130, 120	0.15
[25]		16.0	16.0	160, 170	0.42
[26]		13.4	8.0	240, 390	0.36
[27]		64.0	7.85, 8.10	60	0.15
[28]		0.11	120	70	0.11
[29]	Piezoelectric	1.2	2.1, 1.8	195	0.0082
[30]		0.79	9.3	1370	0.44
[31]		0.50	12.5	900	0.087
This work		36.0	21.6, 19.4	89, 111	0.48

**Figure A1:**
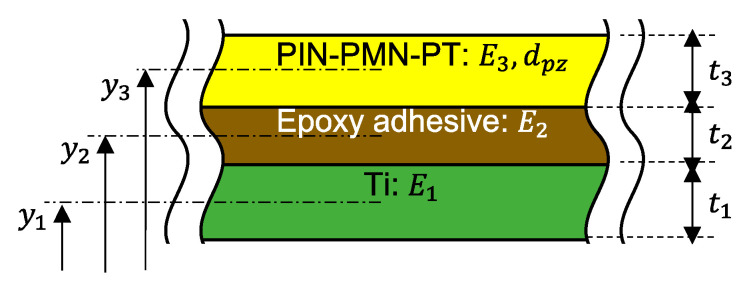
Layer configuration of piezoelectric actuator.

**Figure A2:**
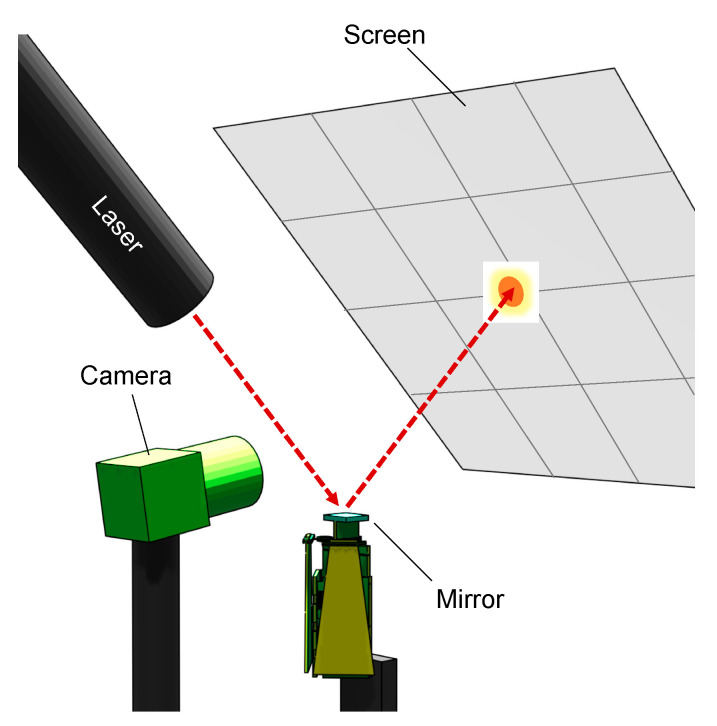
Evaluation setup.

## Data Availability

The data presented in this study are available on request from the corresponding author.

## Appendix A. FEA Model Parameters

Table A1 summarizes the material properties used in the FEA. Piezoelectric actuators and transmission mechanisms are composed of laminated materials. For example, a piezoelectric actuator consists of three layers of titanium, PIN-PMN-PT, and an epoxy layer that bonds these layers, as shown in Figure A1. The calculation method for the bending stiffness of the laminate is described below.

**Table A1:** Material properties used in FEA.

Material	Property	Value
PIN-PMN-PT	Young’s modulus	14.3 GPa ^1^
	Density	8000 kg/m^3^
	Piezoelectric coefficient	−1156 pm/V
Titanium	Young’s modulus	116 GPa
	Density	4500 kg/m^3^
Polyimide	Young’s modulus	9.0 GPa
	Density	1500 kg/m^3^
Silicon	Young’s modulus	160 GPa
	Density	2330 kg/m^3^

^1^ Given by the reciprocal of $s_{22}^{E}$ value of PIN-PMN-PT [33].

First, the position of the neutral axis, $y_{c}$, is calculated using the following equation:

(A1) $$y_{c}=\frac{\sum_{i}E_{i}t_{i}y_{i}}{\sum_{i}E_{i}t_{i}},$$

where $E_{i}$, $t_{i}$ and $y_{i}$ are the Young’s modulus, thickness, and position in the thickness direction of the i-th layer, respectively (as illustrated in Figure A1). The bending stiffness per unit width, s, is given by:

(A2)
$$s=\underset{i}{\sum }E_{i}\left(\frac{t_{i}^{3}}{12}+t_{i}{\left(y_{i}-y_{c}\right)}^{2}\right).$$

The bending moment per unit width, m, caused by the piezoelectric effect with voltage V, is obtained by [38]:

(A3)
$$m=E_{3}d_{pz}\left(y_{3}-y_{c}\right)V.$$

## Appendix B. Hinge Spring Constant and No-Load Deflection Used in the Simplified Theoretical Model

In this section, we summarize the derivation of the properties of hinges and actuators using a simplified theoretical model. First, the PI hinge is simplified to assume that the entire hinge deforms with a constant curvature. The rotational spring constant of the hinge can be obtained as follows:

(A4) $$k=\frac{EI}{L},$$

where E, I and L are the Young’s modulus, cross-sectional secondary moment, and hinge length, respectively.

Next, we consider the spring constant of the trapezoidal-shaped actuator. The width at the root is $w_{r},$ the width at the tip is $w_{t}$, and the length is $L_{A}$. The coordinate z is taken to be in the longitudinal direction, where z=0 is the tip and $z=L_{A}$ is the root. The displacement δ of the actuator when a force F is applied to the tip can be expressed by the following differential equation:

(A5)
$$\frac{\partial^{2}\delta }{\partial z^{2}}=\frac{Fz}{sw\left(x\right)},\;w\left(x\right)=w_{t}+\left(w_{r}-w_{t}\right)\frac{z}{L_{A}}.$$

Solving for this using a boundary condition, ∂δ/∂z=0 and δ=0 at $z=L_{A}$, we obtain:

(A6) $$\delta =\frac{Fz}{s{\hat{\Delta w}}^{2}}\left[\frac{1}{2}\hat{\Delta w}\left(z^{2}-2L_{A}z+L_{A}^{2}\right)+w_{t}\left(z-L_{A}\right)-\frac{w_{t}}{\hat{\Delta w}}\left(\hat{\Delta w}z+w_{t}\right)\ln\left(\frac{w_{t}+\hat{\Delta w}z}{w_{r}}\right)\right],$$

where $\hat{\Delta w}=\left(w_{r}-w_{t}\right)/L_{A}$. The tip displacement can be expressed as:

(A7)
$${\delta |}_{z=0}=\frac{Fz}{s{\hat{\Delta w}}^{2}}\left[\frac{L}{2}\left(w_{r}-3w_{t}\right)-\frac{w_{t}^{2}}{\hat{\Delta w}}\ln\left(\frac{w_{t}}{w_{r}}\right)\right].$$

Thus, the spring constant of the actuator, $k_{A}$ can be given by:

(A8)
$$k_{A}=s{\hat{\Delta w}}^{2}/\left[\frac{L}{2}\left(w_{r}-3w_{t}\right)-\frac{w_{t}^{2}}{\hat{\Delta w}}\ln\left(\frac{w_{t}}{w_{r}}\right)\right].$$

Next, the derivation of the no-load deflection is described. The no-load curvature caused by the piezoelectric effect, κ, is given by:

(A9)
$$\kappa =\frac{m}{s}.$$

Thus, the no-load tip deflection $\delta_{A}$ is obtained by:

(A10)
$$\delta_{A}\left(V\right)=\frac{1}{2}\kappa L_{A}^{2}=\frac{E_{3}d_{pz}\left(y_{3}-y_{c}\right)}{2s}L_{A}^{2}V.$$

## Appendix C. Evaluation Method

Figure A2 shows the evaluation system. A red laser (37-029; Edmund Optics Corp., Barrington, NJ, USA) was used to irradiate the mirror, and the reflected light was received by the screen. A camera (FDR-AX700; Sony, Tokyo, Japan) was used to capture the bright spots on the screen, and the deflection angle of the mirror was estimated from the positional change of the bright spots on the screen. The high-voltage signals used to drive the piezoelectric actuators were generated by a combination of a function generator (4054 B, BK Precision, Yorba Linda, CA, USA) and a high-voltage amplifier (HJPZ-0.3P×3, Matsusada Precision Inc., Kusatsu, Japan).
